# Patch quality and habitat fragmentation shape the foraging patterns of a specialist folivore

**DOI:** 10.1093/beheco/arac068

**Published:** 2022-07-17

**Authors:** Mathew S Crowther, Adrian I Rus, Valentina S A Mella, Mark B Krockenberger, Jasmine Lindsay, Ben D Moore, Clare McArthur

**Affiliations:** School of Life and Environmental Sciences, University of Sydney, Sydney, New South Wales 2006, Australia; School of Life and Environmental Sciences, University of Sydney, Sydney, New South Wales 2006, Australia; School of Life and Environmental Sciences, University of Sydney, Sydney, New South Wales 2006, Australia; Sydney School of Veterinary Science, University of Sydney, Sydney, New South Wales 2006, Australia; Sydney School of Veterinary Science, University of Sydney, Sydney, New South Wales 2006, Australia; The Westmead Institute for Medical Research, 176 Hawkesbury Road, Westmead, New South Wales 2145, Australia; Marie Bashir Institute for Emerging Infectious diseases and Biosecurity, University of Sydney, 176 Hawkesbury Road, Westmead, New South Wales 2145, Australia; School of Life and Environmental Sciences, University of Sydney, Sydney, New South Wales 2006, Australia; Hawkesbury Institute for the Environment, Western Sydney University, Richmond, New South Wales 2753, Australia; School of Life and Environmental Sciences, University of Sydney, Sydney, New South Wales 2006, Australia

## Abstract

Research on use of foraging patches has focused on why herbivores visit or quit patches, yet little is known about visits to patches over time. Food quality, as reflected by higher nutritional quality and lower plant defenses, and physical patch characteristics, which offer protection from predators and weather, affect patch use and hence should influence their revisitation. Due to the potentially high costs of moving between patches, fragmented habitats are predicted to complicate foraging decisions of many animals. We aimed to determine how food quality, shelter availability and habitat fragmentation influence tree reuse by a specialist folivore, the koala, in a fragmented agricultural landscape. We GPS-tracked 23 koalas in northern New South Wales, Australia and collated number of revisits, average residence time, and average time-to-return to each tree. We measured tree characteristics including food quality (foliar nitrogen and toxic formylated phloroglucinol compounds, FPCs concentrations), tree size, and tree connectedness. We also modeled the costs of locomotion between trees. Koalas re-visited isolated trees with high leaf nitrogen disproportionately often. They spent longer time in trees with high leaf nitrogen, and in large trees used for shelter. They took longer to return to trees with low leaf nitrogen. Tree connectivity reduced travel costs between patches, being either individual or groups of trees. FPC levels had no detectable effect on patch revisitation. We conclude that food quality and shelter drive koala tree re-visits. Scattered, isolated trees with nutrient-rich leaves are valuable resource patches for koalas despite movement costs to reach them.

## Teaser

We GPS-tracked Australia’s iconic koalas within a fragmented agricultural landscape to determine why they would return to the same trees or groups of trees. We found that they selected trees with high nitrogen, and larger trees that provided shelter from the heat. This often involved moving large distances across a hostile cleared landscape. Hence, scattered trees on farms, especially when the trees are of high quality, are essential for arboreal animals.

## INTRODUCTION

Foraging herbivores face the dietary challenge of how to maintain an adequate nutrient intake while avoiding excessive costs of plant defenses (McArthur et al. 2014). Specialist folivores in particular, must often cope with diets that offer low availability of essential nutrients, such as protein and amino acids, yet impose high metabolic costs associated with ingesting plant secondary metabolites (PSMs) (Marsh et al. 2007). PSMs such as tannins can bind to protein, reducing their digestibility, and toxins can cause physiological damage (McArthur and Sanson 1991; Sorensen et al. 2004; Shipley et al. 2012). Plant toxins affect herbivore feeding behavior by limiting their food choices (Provenza and Malechek 1984; McArthur et al. 1993) and reducing food intake (Wiggins et al. 2003; Sorensen et al. 2004; Marsh et al. 2005; Moore and Foley 2005), by limiting meal size and increasing the time between feeding bouts (Wiggins et al. 2003, 2006a, 2006b). The ability to cope with plant toxins differs between herbivore species. Specialist folivores have a higher tolerance for some PSMs than generalists because they have evolved more efficient pathways to neutralize plant toxins (McArthur and Sanson 1991; Shipley et al. 2012), but they may still have to reduce food intake once toxin thresholds are reached. Consequently, nutrients may attract herbivores to food patches, while PSMs may repel them.

Nutritional quality and PSM concentrations vary within patches of trees, even of a single species, and across the landscape (Moore et al. 2010). In such heterogeneous landscapes, herbivores are predicted to prefer to visit high-quality food patches, e.g., high in nutrients with low plant defenses. Consistent with this prediction, free-ranging pygmy rabbits (*Brachylagus idahoensis*) and greater sage grouse (*Centrocercus urophasianus*) both select for patches with low plant toxin concentrations (Frye et al. 2013; Ulappa et al. 2014). Herbivores also must decide when to quit food patches. Staying longer in a patch can provide greater nutrient intake but may incur increasing costs associated with metabolizing ingested toxins or increasing risk of predation (Kotler et al. 1991; Mella et al. 2015). Manipulative experiments have demonstrated the effects of plant nutrition and toxins on patch quitting (Nersesian et al. 2011; McArthur et al. 2012; Bedoya-Pérez et al. 2014). For example, common brushtail possums (*Trichosurus vulpecula*) spend more time feeding in safe patches low in toxins and switch to riskier patches when toxins levels are higher in the safe patches (Nersesian et al. 2011).

Foraging is not the only motivation for using habitat patches; shelter can also be important. Herbivores may use shelter to avoid predators or to thermoregulate (Brown et al. 1988; Crowther et al. 2014). As a result, herbivores can have different habitat requirements (for food or shelter) within the diel cycle, driving the differential use of patches (Owen-Smith et al. 2010). In environments with large differences between diurnal and nocturnal temperatures, herbivores may forage more often during cooler times of the night and seek shelter during periods of heat. During summer, Beira antelopes (*Dorcatragus megalotis*) use patches associated with increasing tree cover and are more active foraging at night (Giotto et al. 2013). Similarly, bridled nail-tail wallabies (*Onychogalea fraenata*) and red-necked wallabies (*Macropus rufogriseus*) shelter in areas with high plant cover which are not just food plants (Fisher 2000; le Mar and McArthur 2005).

Habitat fragmentation complicates the foraging decisions faced by herbivores. Greater fragmentation likely increases movement costs between foraging patches for many species, either because individuals must travel greater distances, increasing the energetic costs, or because they must move through open habitat, often increasing predation risk. Consistent with this, predation rates were higher in root voles (*Microtus oeconomus*) occupying fragmented habitats with long interpatch distances (Andreassen and Ims 1998). Furthermore, when fragmentation isolates habitat patches, herbivores may have fewer food choices within patches and so need to move more between patches to satisfy their needs. For example, the common brushtail possum, a generalist browser, incurred a four-fold increase in travel costs to switch between food sources and so overcome effects of PSMs when the foraging landscape was manipulated (Wiggins et al. 2006b).

While many studies focus on questions of why herbivores visit or quit patches (Mella et al. 2018b), an equally important question is why herbivores return to previously visited patches. Incorporating metrics on revisits to patches in studies of foraging, space use and animal movement adds a temporal component to the cost/benefit analysis that studies on visits per se cannot achieve. In doing so, we are better able to understand and measure the long-term quality of food patches from the herbivore’s perspective. Since the quality and abundance of resources can vary across the landscape, individuals may return and spend more time in more favorable areas. The return of animals to previously visited patches is known as recursion (Riotte-Lambert et al. 2013; English et al. 2014; Van Moorter et al. 2016); and recursions are likely to be an important foraging strategy for herbivores to exploit high-quality patches (McNaughton 1985; Bar-David et al. 2009).

Studies examining patch recursions are few (Riotte-Lambert et al. 2013), but a similar process known as “traplining” has been studied in insects, birds and primates (Berger-Tal and Bar-David 2015). For example, hummingbirds often return to previously visited flowers in regularly repeated, ordered steps that optimize intake of nectar (e.g., Gill 1988). Few studies have examined the temporal use of patches by individuals in relation to food quality and shelter in natural environments. In two large herbivores, the Asiatic wild ass (*Equus hemionus*) and red deer (*Cervus elaphus*), number of revisits to foraging sites increased during periods of increased plant productivity (Giotto et al. 2015; Seidel and Boyce 2015) suggesting some form of harvest strategy. In mountain gorillas (*Gorilla gorilla beringei*), frequency of revisits was greater to areas with abundant high-protein food (Watts 1998). Such studies provide an understanding of factors driving the temporal foraging patterns of herbivores and re-use of patches.

Our aim was to determine how the temporal use—and particularly the re-use—of trees by a specialist arboreal folivore, the koala (*Phascolarctos cinereus)*, is driven by plant chemistry, shelter and habitat fragmentation in an agricultural landscape. We used the koala as our model species because it is a specialist herbivore that depends entirely on trees for food and shelter (Ellis et al. 2002, 2009; Pfeiffer et al. 2005; Matthews et al. 2007; Crowther et al. 2014). Its specialized diet, primarily of *Eucalyptus* leaves, offers low nutrient concentrations and can be high in toxins and tannins (Eschler et al. 2000; Moore and Foley 2005). Toxins, such as formylated phloroglucinol compounds (FPCs), can be a strong feeding deterrent in captive individuals (Marsh et al. 2007) and influence tree selection by free-ranging koalas (Moore and Foley 2005; Marsh et al. 2014; Stalenberg et al. 2014). Koalas can move between trees when canopies overlap, but generally move on the ground between trees, and have no choice when a fragmented habitat isolates forest and woodland patches or individual trees. Leaf chemistry affects the number of koala visits to particular individual trees (Moore and Foley 2005)) and time spent feeding in trees (Marsh et al. 2014), and in some cases koalas have been observed to return frequently to previously used trees (Hindell et al. 1985; Mitchell 1990; Matthews et al. 2007). Water requirements may also affect tree re-use by koalas, as leaves higher in moisture are preferred (Munks et al. 1996; Ellis et al. 2002, 2010; Wu et al. 2012). Koalas repeatedly visit artificial water stations when moisture available in leaves is not sufficient to meet their water balance needs (Mella et al. 2019), suggesting re-use of previously visited patches is a strategy utilized by koalas when resources are valuable. How tree quality and habitat fragmentation affect decisions of when and how often individual koalas return to previously used trees is unknown. Yet, understanding patterns of tree use by individuals provides greater predictive capacity for defining habitat quality than understanding general patterns at the population level.

We hypothesized that factors influencing koala revisits to resource trees would differ between day and night, as koalas are predominately nocturnal (Ellis et al. 2009; Marsh et al. 2014). We predicted that during the night, koalas would revisit trees as a function of food quality, more often revisiting trees high in leaf nitrogen and low in FPCs. We also hypothesized that tree connectivity would affect the temporal use of patches, defined as individual or close grouping of trees (Rus et al. 2021) because of movement costs. We predicted that koala revisits would decrease (fewer revisits and less frequently) to trees that were less connected within the landscape. We predicted that when choosing trees for daytime use, koalas would revisit trees that provide more shelter (as reflected in foliar cover) from daytime temperatures.

We analyzed movement patterns to determine the re-use of patches by measuring the number of revisits, average time spent in trees (residence time [RT]), and average time-to-return (TtoR). For habitat fragmentation, we measured the connectivity of habitat surrounding the used trees. We used least-cost paths (LCP) (Etherington and Holland 2013) as an index of the travel costs individuals experience when revisiting trees and linked it to habitat fragmentation. The least-cost paths were based on open vs. forested ground and on the slope of the terrain.

## MATERIALS AND METHODS

### Study area

We conducted the study on the Liverpool Plains near the town of Gunnedah, north-eastern New South Wales (NSW), Australia (30°59ʹS, 150°16ʹE). The landscape is a matrix of productive agricultural land and isolated open woodland patches, comprising *Eucalyptus* species including white box *E. albens*, yellow box *E. melliodora*, poplar box *E. populnea*, and river red gum *E*. *camaldulensis* (Lunney et al. 2012; Crowther et al. 2014; Dargan et al. 2019).

### Koala tracking

We used movement data from 23 out of 58 koalas fitted with GPS collars (average mass 120 g; Sirtrack, Hawkes Bay, New Zealand), between October 2015 and November 2017 (Crowther et al. 2021; Rus et al. 2021). Movement data ranged from April to August (non-breeding season) in 2016, and September to February (breeding season) in 2015 and 2017 (Table 1). Although patch revisitation is likely to differ between sexes, and between season (Ellis et al. 2009; Rus et al. 2021), there was insufficient power to test these effects, The GPS collars recorded koala positions every 4 h for up to 5 months. For consistency, we only included 4 months of movement data from each individual koala for our analysis. We determined the accuracy of the GPS collars using a static test next to a trigonometric survey mark in Gunnedah (31° 01ʹ 47.05717″ S, 150° 16 ʹ 04.32014″ E (Crowther et al. 2014; Rus et al. 2021). The distance root mean square error was calculated to be 9.05 m.

**Table 1:** Year tracked and sex of GPS-collared koalas used in the study

Year	Number of koalas	
	Females	Males
2015	5	5
2016	6	4
2017	2	1

### Koala tree use

We quantified three metrics from movement data to describe tree use by koalas: 1) number of visits to each tree, 2) average residence time (RT), and 3) average return time to each tree (TtoR). We used ArcMap to create 5 m buffers around the sampled trees, identified through koala tree use, which were used to analyze the level of tree use by each koala. We considered a koala was using a tree when it was within the tree buffer for at least two consecutive location records (i.e., ≥ 4 h), and this was defined as a visit. If an animal left the tree buffer for more than two consecutive location records, it signified the end of the visit. For each sampled tree, we determined the number of revisits by counting the number of visits by a particular koala. Residence time was calculated as the average visit duration, and the TtoR was the average time between each visit.

### Tree sampling

We used the location data collected by the GPS loggers to identify trees that were visited by koalas. These trees were located using a handheld GPS unit (Garmin eTrex10), with accuracy less than 3 m and mostly within 1 m, due to the open canopy cover of agricultural landscapes. Due to the large spacing of trees across the site, most sampled trees were more than 10 m from their conspecifics, reducing the uncertainty (due to GPS error) of the true tree use by koalas. We measured physical attributes of the trees: tree height (m), diameter at breast height (~137 cm from ground level; in cm), and relative foliar shelter cover (divided into low, medium and high categories) (Crowther et al. 2014). Tree height was determined using a range finder by measuring the distances to the top and bottom of the tree forming a right triangle between the observer and the tree. We used the following formula to determine tree height: $tree\;\;\;height=\frac{{\left(top\;distance\right)}^{2}}{\left(base\;distance\right){!}^{2}}+observer\;height.$ Foliar projection cover of each tree was assessed visually, based on methods by Crowther et al. (2014). To determine the leaf chemistry of each tree, we sampled approximately 50 g of mature leaves from the lower third of each tree canopy, using pole-mounted pruners or by pulling a branch from the tree using a line launched from a Big Shot^TM^ throwbag launcher. Leaves were placed in zip-lock bags and into a portable freezer at −20 °C. Leaves were subsequently freeze-dried and ground in a Foss Cyclotec 1093 mill (ANKOM Technology, Macedon, New York) using a 1 mm screen. The dried leaves were stored in the dark in polyethylene vials at room temperature for later analysis.

### Leaf chemistry

Near-infrared spectroscopy (NIRS) is a non-destructive technique for analyzing large numbers of samples (Foley et al. 1998). A small subset of samples can be selected that represent the chemical variation of all samples taken, to perform time-consuming “wet” chemistry. Principal components analysis (PCA) is used to detect the variability within the dataset and determine outliers (Towett et al. 2013), which are then automatically removed. We performed wet chemistry to determine the true values for each constituent (described in detail below). The data set was then split into two sets, a calibration set for determining the model, and a smaller validation set to test the model (approximately 10% of the data set). These models were then used to predict the chemistry of the entire sample set and only predicted values were used for statistical analysis. Calibrations were developed using partial least squared models with cross-validation and subsequently used to determine formylated phloroglucinol compounds (FPC) and N concentrations following Moore et al. (2005).

We measured the foliar concentration of FPCs (mg/g DM) in 220 samples and total nitrogen (% DM; hereafter referred to as N) in 412 samples. The spectra of all the dried, ground leaf samples were collected from 400 to 2500 nm using a Near-Infrared Spectrometer (FOSS XDS rapid content analyzer). We also measured digestible Nitrogen, although it was strongly correlated with total Nitrogen, and had a lesser fit to the models, so it was excluded from further analyses.

FPC concentrations were determined using high-performance liquid chromatography (HPLC). Approximately 50 mg ± 1 mg of ground leaf sample was added to 4 mL of extraction solvent (HPLC grade acetonitrile containing 0.1% trifluoroacetic acid) and sonicated for 2 min (Wallis and Foley 2005). Samples were then filtered (0.25 µm nylon filter) into an autosampler vial and then loaded into the Agilent 1200 series HPLC. We injected 10 μL onto the HPLC and analyzed it on a Wakosil GLC18RS column maintained at 37 °C with a flow rate of 0.75 mL min^-1^. Extracts were eluted under six gradient conditions using 0.1% trifluoroacetic acid (TFA) in acetronitrile and 0.1% TFA in Milli-Q water and held for 3 min. The specific FPC compounds present in each sample were quantified using standard curves developed from authentic standards and identified by their unique retention time (min) when they came off the column. We measured the peak response of 18 major peaks on a UV detector at 275 nm from the resulting chromatographs. N concentrations of 200 mg samples of freeze-dried ground leaf were determined using an automated Dumas combustion method on a Leco TruMac carbon/nitrogen analyzer (Leco Corporation, USA).

In this landscape, koalas show seasonal patterns of use of artificial water drinkers, with more visits in summer than other seasons (Mella et al. 2019), indicating both the value of water to them and the differential value over time. It is therefore possible that variation in leaf water content, if large enough among individual trees, may contribute to patterns of tree use and re-use of trees by koalas. We could not test this here because the spread of our data per koala (varying across time) meant we could not adequately incorporate the differential use of water across seasons seen with drinkers into the analysis.

### Tree connectivity and least-cost paths

We used tree connectivity to assess the level of habitat fragmentation surrounding trees revisited by koalas, following Rus et al. (2021). When habitat is more fragmented, trees are less connected to each other. To quantify tree connectivity, we created a 50 m buffer around each sampled tree and calculated the amount of treed habitat using the 2011 NSW woody vegetation extent layer (i.e., trees > 2 m in height; Office of the Environment and Heritage, NSW, Australia). The buffer distance of 50 m was used because it represented the average daily distance movement by koalas at our study site. We selected four metrics in FRAGSTATS (McGarigal and Marks 1995) associated with patch aggregation across the 50 m buffer centered on each focal tree, 1) Clumpiness index (Clumpy), 2) Perimeter-Area Fractal Dimension (PAFRAC), 3) Cohesion, 4) Aggregation Index (AI). *CLUMPY* represents the spatial organization of patches and measures how far they deviate from a spatially random distribution. *PAFRAC* is a measure of shape complexity, with less complex shapes having simple perimeters and more complex shapes being convoluted. *Cohesion* measures the physical connectedness of the corresponding patch type and increases as the patch type becomes more aggregated in its distribution; hence, more physically connected. *AI* measures the frequency with which different pairs of patch types (including like adjacencies between the same patch type) appear side-by-side on the map and it increases as the focal patch type is increasingly aggregated and equals 100 when the patch type is maximally aggregated into a single, compact patch (for more information, see McGarigal and Marks 1995).

We were interested in determining the movement cost for koalas revisiting trees that were more isolated. Estimation of movement costs was based on cost surfaces, which are representations of cost related to energy expenditure and predation risk. We used ArcMap 10.5 (ESRI, Redlands, CA) to create cost surfaces to determine the least-cost paths (LCP) and the accumulated costs for each sampled tree. Cost surfaces represent the level of landscape resistance that an animal may experience as it moves from one location to another. LCPs are the routes of maximum efficiency from one location to another as a function of the distance traveled and the costs traversed (Etherington and Holland 2013). Least-cost paths can be highly sensitive to the entered resistance values, which can affect the accumulated cost values (Etherington and Holland 2013). Resistance values are normally inferred from species biology and movement across the landscape (e.g., Jeliazkov et al. 2019). Since there was no information on the costs of movement by koalas, we used the movements of five dispersing koalas to determine the resistance values. We chose a resistance value of one as the base cost of koalas moving through tree habitat for both cost surfaces, and either two (cost surface A) or eight (cost surface B) for open ground habitat. A cost of two would represent double the cost for koalas moving across open habitat and eight would mean eight times the cost of movement. We also included the costs of koalas moving over sloped terrain using a 5 m resolution digital elevation model. After modeling the LCP for the two cost surfaces, we compared the path distances with the observed movement by the dispersing koalas (see Supplementary Material). We found that cost surface two was closer to the observed values. Therefore, we used the resistance values from the cost surface A to calculate the least-cost for the sampled trees. We modeled koalas moving from the edge of the 50 m buffer to the center (location of sampled tree).

### Statistical analysis

R version 3.6.1 (R Development Core Team 2019) was used for all statistical analysis. We compared the concentrations of N and FPCs between the different tree species using a one-way ANOVA, after confirming diagnostic plots satisfied assumptions of the parametric test.

Generalized mixed models were used to determine whether tree characteristics (physical and chemical) and/or connectivity could explain intensity of tree use by koalas (i.e., number of revisits, RT, and TtoR). We used mixed models to account for the repeat visits to each tree, including the individual animal as a random factor. Diagnostic plots of our data showed non-normal distributions, and to overcome this, we used a Gamma distribution with a log-link for RT and TtoR, and a Poisson distribution with a log-link for number of revisits. We created sets of models combining the independent variables (i.e., DBH, Canopy Cover, leaf N, tree connectivity, and tree height), with the measures of intensity of tree use as the dependent variables. FPCs were not included due to their low concentrations. We ranked the models using the adjusted Akaike Information Criterion (AIC_c_) (Burnham and Anderson 2002). We then examined the top models based on the ΔAICc, log-likelihood and Akaike weights (*w*_*i*_) and then calculated model-averaged estimates and standard errors for models < 2 ΔAICc (R package “MuMln” ver. 1.42.1; (Bartoń 2020).

We used linear models to determine the effect of tree connectivity (Cohesion) on the cost of movement (i.e., accumulated cost) to revisited trees. We applied a fourth root transformation on the response variable to satisfy the requirements of linear models.

## RESULTS

### Tree species selected by koalas

*Eucalyptus populnea* accounted for 67% of the trees selected by koalas, with *E. albens* (14%)*, E. camaldulensis* (9%), *E. conica* (3%)*, E. dealbata* (11%), *E. largiflorens* (<1%), and *E. melliodora* (5%) making up the remaining 33%. Previous transect surveys (Dargan et al. 2019; Crowther et al. 2021) have indicated that the proportions of each eucalypt are *E. populnea* (32%), *E. albens* (1436%)*, E. camaldulensis* (7%), *E. conica* (<1%)*, E. dealbata* (13%), *E. largiflorens* (<1%), and *E. melliodora* (4%).

### Comparison of leaf chemistry between tree species

**Figure 1:**
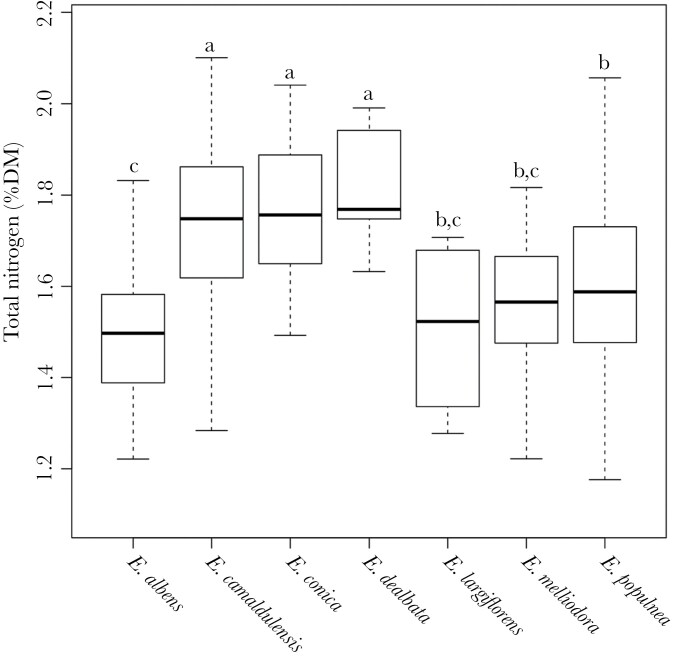
Comparison of total leaf nitrogen by *Eucalyptus* species sampled across koala home-ranges. Letters were generated for each *Eucalyptus* species, and species sharing the same letter are not significantly different. Boxplots show median (middle horizontal lines), first and third quartiles (box hinges), data range (whiskers). Sample sizes were *E. albens* (*n* = 57)*, E. camaldulensis* (*n* = 24), *E. conica* (*n* = 11)*, E. dealbata* (*n* = 6), *E. largiflorens* (*n* = 6), *E. melliodora* (n = 17), and *E. populnea* (*n* = 199).

**Figure 2:**
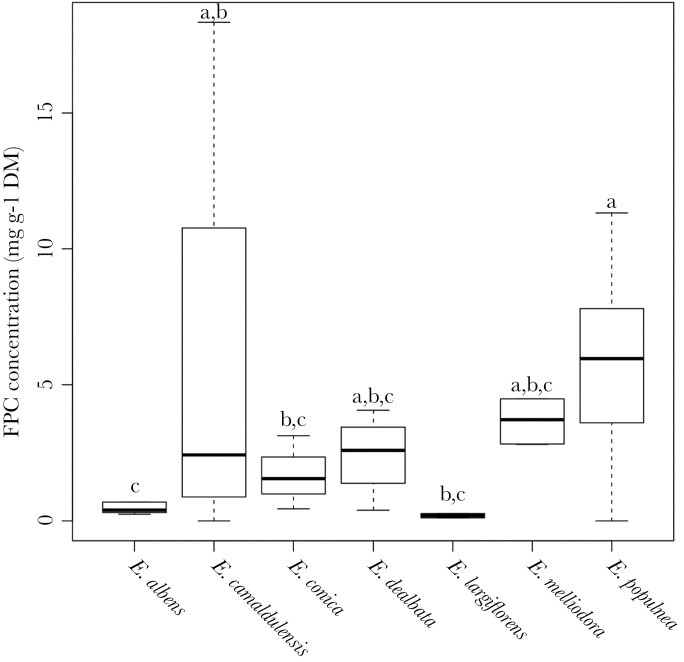
Comparison of FPC concentrations by *Eucalyptus* species sampled across koala home-ranges. Letters were generated for each *Eucalyptus* species, and species sharing the same letter are not significantly different. Boxplots show median (middle horizontal lines), first and third quartiles (box hinges), data range (whiskers). Sample sizes were *E. albens* (*n* = 13)*, E. camaldulensis* (*n* = 24), *E. conica* (*n* = 4)*, E. dealbata* (*n* = 4), *E. largiflorens* (*n* = 3), *E. melliodora* (*n* = 5), and *E. populnea* (*n* = 73).

There was a significant effect of tree species on leaf N concentration (*F*_*6,405*_ = 10.2, *P* < 0.001) and FPC concentration (*F*_*6,213*_ = 6.1, *P* < 0.001). Concentration of N was significantly higher in *E. dealbata* than both *E. populnea* and *E. albens* (Figure 1). FPC concentration in *E. populnea* was significantly higher than in *E. albens, E. conica*, and *E. largiflorens* (Figure 2).

### Night tree use

**Figure 3:**
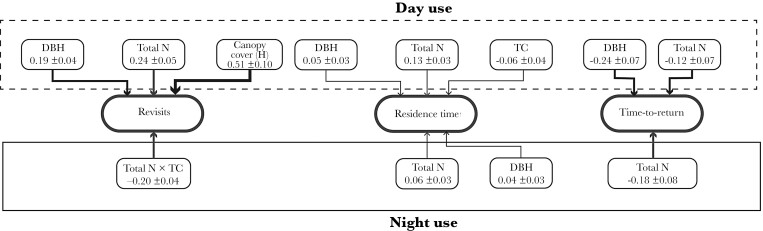
Path diagrams showing the effect of the most important variables explaining koala tree use: number of revisits, residence time and Time-to-Return. Line width is weighted by the model-averaged standardized parameter values of the mixed-effects models, with width increasing with increasing parameter estimate values. Text boxes include the variable names and standardized parameter estimate values with standard errors. Dashed box represents day tree use by koalas and solid box night use. TC = measure of tree connectivity based on cohesion metric; DBH = tree diameter at breast height; Total N = total leaf nitrogen.

**Figure 4:**
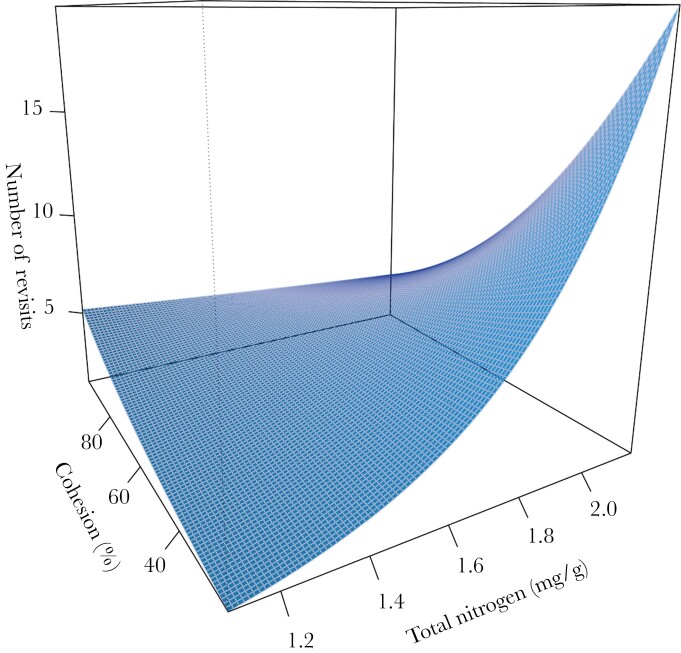
Interaction plot representing the number of tree revisits during the night by koalas as a function of the interaction between total concentration of leaf nitrogen and cohesion. Higher cohesion represents higher tree connectivity.

**Figure 5:**
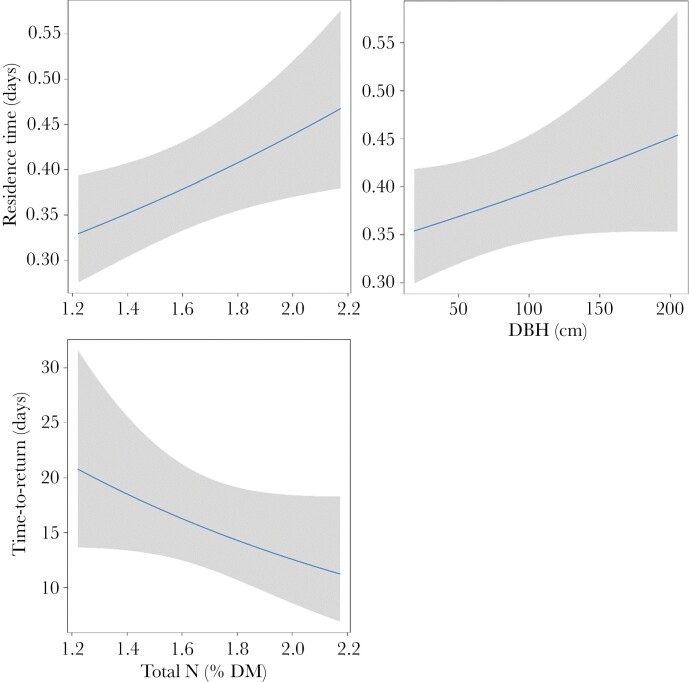
Plots representing the effects of N and DBH on the average Residence Time and average Time-To-Return for trees during nightly revisits by koalas. Solid line represents the predicted relationship, and shaded area represents 95% confidence interval for the prediction.

**Figure 6:**
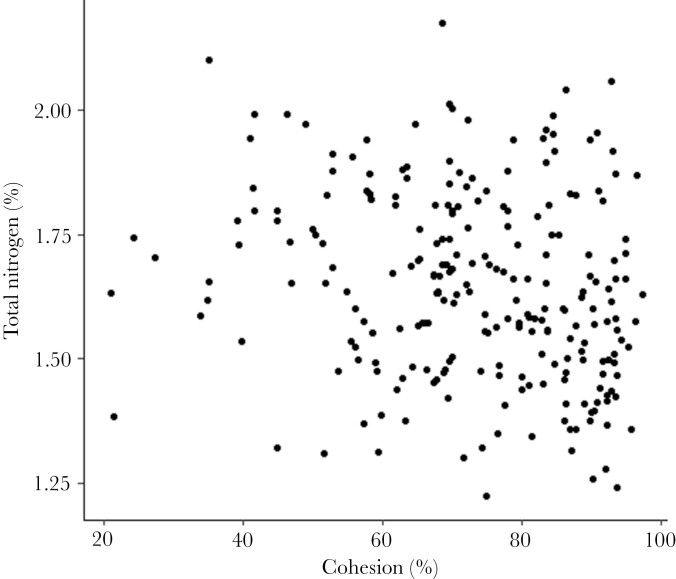
Plot showing the relationship between cohesion (%) and Total Nitrogen (%).

For the number of revisits to trees by koalas, there was an interactive effect of leaf N and tree connectivity (Figure 3). Koalas made substantially more revisits to trees with greater leaf N at lower cohesion, yet slightly more revisits to trees with less leaf Nitrogen at higher cohesion (Figure 4). Average time in trees (RT) was influenced by DBH and leaf Nitrogen (Figure 3). Koalas spent more time in larger trees, and trees that contained high concentrations of Nitrogen (Figure 5). The average TtoR (Time to Return) was only influenced by N, with koalas returning much faster to trees that were high in N than those low in N (on average every 2 vs. every 3 weeks, respectively, Figure 5). Some koalas with adjacent home ranges used the same individual trees, but none used the same individual trees at the same time. There was also no relationship between cohesion and total Nitrogen (Figure 6).

### Day tree use

**Figure 7:**
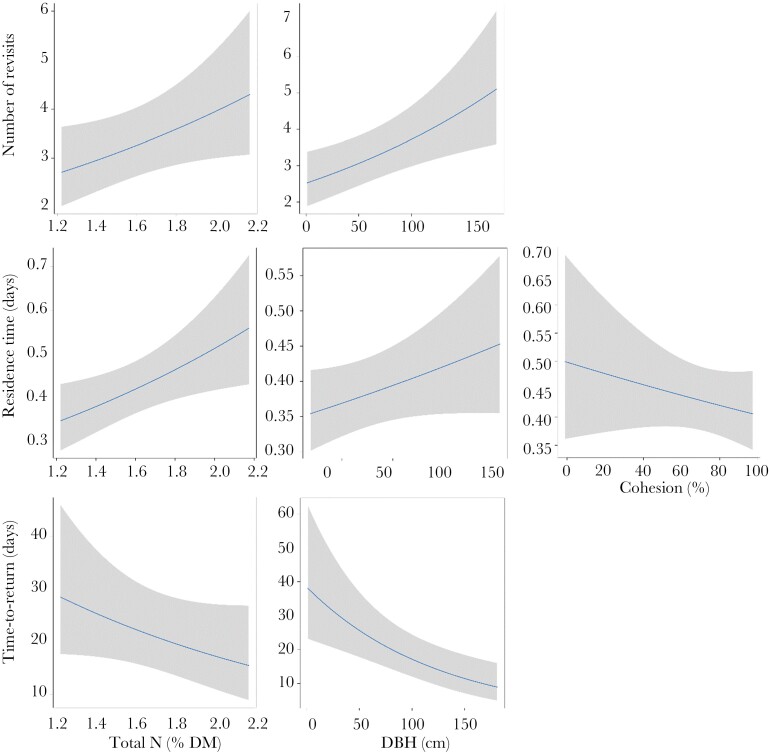
Plots representing the effects of N, DBH, and cohesion (measure of tree connectivity) on the number of revisits, average Residence Time, and average Time-To-Return for trees during day-time revisits by koalas. Solid line represents the predicted relationship, and shaded area represents 95% confidence interval for the prediction.

DBH, Total N, and tree foliar cover within species were the best predictors for koala tree revisits during the day (Figure 3). Koalas revisited large trees, trees that contained high leaf nitrogen, and trees that had more foliar cover. Koalas also spent more time in large trees, trees that were less connected, and trees that contained high concentrations of N (Figure 7). The average TtoR was negatively influenced by DBH and Nitrogen (Figure 3). Koalas returned sooner (smaller TtoR) to trees that were larger and trees that were high in N (Figure 7). There was no significant relationship between FPC concentration and TtoR. Some koalas with adjacent home ranges used the same individual trees, but none used the same individual trees at the same time.

### Least-cost paths

**Figure 8:**
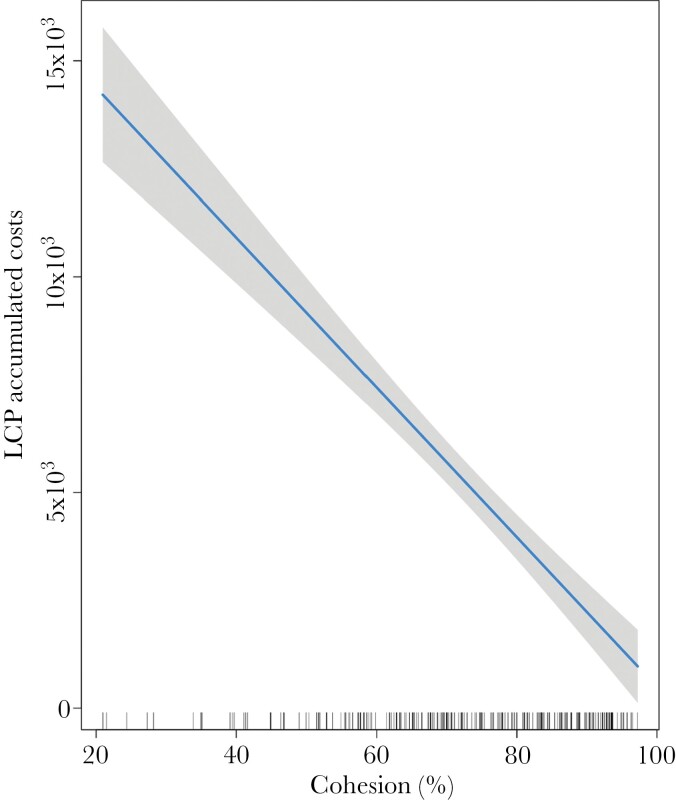
The effects of cohesion (measure of tree connectivity) on the accumulated costs modeled by the least-cost paths (LCP). Solid line represents the predicted relationship, and shaded area represents 95% confidence interval for the prediction.

There was a significant relationship between tree connectivity, measured by the cohesion metric, and the accumulated cost of Least-Cost Path (β = −0.078, SE = ±0.008, *P* < 0.001, *r*_*adj*_ = 0.42). There was an over threefold change in the accumulated costs of koala movement to revisited patches between the highest and lowest levels of tree connectivity (Figure 8).

## DISCUSSION

Whether to visit or quit a patch, as determined by patch quality, has been well-studied for many species of herbivores (Frye et al. 2013; Bedoya-Pérez et al. 2014; Mella et al. 2018b). However, our understanding of the factors contributing to whether an herbivore revisits a patch is limited. The factors driving patch revisitation become even more crucial within a fragmented landscape, particularly when patches vary in quality and costs of movement are high due to predation and energetic costs. Here, we quantified both the physical and nutritional attributes driving a specialist herbivore to revisit patches within a fragmented landscape.

Foliar nitrogen was a strong driver causing koalas to revisit foraging trees. Habitat fragmentation also had a significant impact on their foraging decisions. During the night, there was a much larger increase in the number of revisits to trees with low tree connectivity (i.e., higher habitat fragmentation) provided they were high quality (high in leaf N). This result does not exclude the use of isolated trees with low Nitrogen levels as “stepping-stones” to other patches, as seen in other studies of koala movement in patchy landscapes (Barth et al. 2020; Gallahar et al. 2021), rather it does emphasize the value of isolated trees with high Nitrogen levels for food. In contrast to this, when trees were highly connected, high leaf nitrogen did not result in more frequent revisits. From an individual koala’s perspective, trees that were higher in nitrogen were clearly highly valued, even if they were isolated. Since individual koalas had established home ranges, they may revisit these isolated yet valuable trees as a form of resource defense to deter competitors. Similarly, pied wagtails (*Motacilla alba*) defend their territories against invaders by systematically revisiting foraging spots across their home range, making it harder for invaders to forage on the most valuable areas (Davies and Houston 1981). The contrasting pattern of reduced revisits by koalas to high quality (high leaf N), highly connected, trees may simply be due to availability of more foraging options within close proximity.

The exceptionally high rate of revisits to high quality trees that are less connected to their conspecifics must pose costs to individual koalas, as both energetic costs and risks associated with moving. Yet koalas were willing to incur these costs. A low cohesion index (measure of tree connectivity across the landscape) for a focal tree means that the surrounding trees are far apart, and tree canopies are not interlocking. In such cases, koalas need to move on the ground; and this movement across cleared habitat represents a higher risk of mortality from terrestrial predators such as wild dogs and from roadkill.

We predict that repeated use of less connected nutritious trees could lead to greater browsing pressure, possibly making them vulnerable to reduced growth rate and premature death: a longer-term consequence of habitat fragmentation. Koalas are known to defoliate highly preferred trees while leaving others untouched (Hindell et al. 1985; Menkhorst 2008), and defoliation and death of vast tracts of eucalypts by koalas occurs in the state of Victoria, Australia, where much higher population densities are common (Hindell et al. 1985; Menkhorst 2008). Koalas on the Liverpool Plains are in lower densities than in Victoria (Crowther et al. 2021), and especially after population decline due to drought and chlamydiosis (Lunney et al. 2012; Fernandez et al. 2019; Mella et al. 2019) and hence could explain that there was no evidence of koalas defoliating trees. While koalas may be predicted to use fewer trees if these trees have high Nitrogen content; in the fragmented landscape of the Liverpool Plains, the amount of fragmentation of the patches is the main predictor of home-range size in the area (Rus et al. 2021).

We also found leaf nitrogen played a role in how much time koalas spent in a tree (RT) and how quickly they returned (TtoR). Foragers take advantage of higher-quality patches by increasing their food intake, and koalas do eat more when they spend more time in more nutritious trees (i.e., high nutrients and low toxin; Marsh et al. 2014). Koalas may also revisit trees to monitor leaf renewal rates; returning more often to high leaf N trees to do so. Marsh et al. (2014) speculated that koalas might visit unpalatable (low in N and high in FPCs) trees to update their knowledge about nutritional quality. Leaf quality can fluctuate across time, for example in and out of seasonal or rainfall-driven leaf flush (Pook et al. 1997; Duursma et al. 2016) and as leaves age (Loney et al. 2006). Koalas may therefore optimize their foraging by revisiting trees based on the elapsed time since last visit, although frequent revisits to profitable areas can increase foraging efficiency independent of food renewal rates (Pyke et al. 1977). For example, ovenbirds (*Seiurus aurocapillus*) revisited artificial patches with higher density of food more frequently than lower food density patches when renewal rates were similar (Zach and Falls 1976). Koalas selected more *E. populnea* and *E. albens*, which were variable in total Nitrogen levels, but were also much more represented in the landscape than the other species of *Eucalyptus* (Dargan et al. 2019). Hence koalas select for total Nitrogen levels than they do for tree species.

Contrary to our predictions, plant toxin concentration was not a strong predictor of tree revisits by koalas. This is probably because FPC concentrations (0–15 mg/g) were much lower than previously reported in other eucalypt populations (which were sometimes greater than 50 mg/g) (Moore et al. 2004, 2005; Moore and Foley 2005). *Eucalyptus* leaf FPC, nitrogen, phenolic and terpene concentration can all vary as a function of both genetics and the phenotypic influence of fertilizer (McArthur et al. 2003; O’Reilly-Wapstra et al. 2005; Loney et al. 2006) and drought can also lower concentrations of FPCs in *Eucalyptus* seedlings (McKiernan et al. 2015). The low FPC concentrations of the trees may therefore arise from both genetics and as a phenotypic response to this highly fertile agricultural landscape (Crowther et al. 2009). This may have also be the case for total Nitrogen being a stronger predictor of koala tree-use than digestible Nitrogen, as the fertile soils of the Liverpool Plains also kept phenolics the leaves at low levels.

Leaf water content may also be a driver of tree use in certain seasons, if it varies considerably among trees (Ellis et al. 1995, 2010), given the seasonal patterns in use of artificial water drinkers by koalas in the same landscape (Mella et al. 2019). As our method meant we collected leaves during different times of the year to when the koalas used these trees, we could not adequately incorporate the differential use of water contents of the leaves into the analysis.

Tree canopy cover and DBH had a strong effect on number of koala revisits during the day. Our results show that koalas placed a higher value on trees that provided more shelter (i.e., tree canopy cover), because they returned more often. This is consistent with previous findings in which koalas used trees with higher canopy cover during the day (Ellis et al. 2002, 2009; Crowther et al. 2014; Rus et al. 2021). During high daily temperatures, koalas use large trees to dissipate heat through conduction with the tree limbs (Briscoe et al. 2014). Interestingly, average time spent in trees (RT) and average return time (TtoR) during the day were not affected by tree canopy but by the size of the tree (DBH) and its quality (leaf N). Since large trees are favored by koalas for both food and shelter, koalas might revisit trees that serve both as a foraging patch during the night and shelter during the day. Such large trees with dense canopies therefore represent a valuable resource for koalas, especially in fragmented habitats, because they can provide both food and shelter.

Predation may affect habitat use of koalas (Lunney et al. 2007; Beyer et al. 2018). However, dingoes (*Canis dingo*) and domestic dogs (*C. familiaris*) are not predators of koalas in the study area and although foxes (*Vulpes vulpes*) co-occur with koalas in the region (Mella et al. 2018a), there is no evidence of predation on koalas. Road traffic has a major impact on koala mortality in other parts of its range (Lunney et al. in press) but this study was conducted on farmland where vehicle traffic is low, so it was not considered a major driver of habitat use.

In this study, we integrated movement to explore the ecological question of why individual koalas revisit resource patches. At night, when koalas are actively feeding, revisits to trees were predominantly influenced by leaf nitrogen and tree connectivity, and during the day revisits were influenced by leaf nitrogen and shelter. Koalas revisited isolated trees high in leaf nitrogen much more frequently than more connected trees, possibly to defend such valuable resources from conspecifics. Longer term, frequent returns may lead to the demise of these isolated trees through browsing. Loss of such valuable trees, for any reason, will further fragment habitat. We conclude, therefore, that it is important to further protect high (nutrient) quality, isolated trees and replant patches of trees to increase their connectivity with others, and to replace older dying trees. Further research is needed to better understand the effects of increased herbivory on isolated resource patches and their effects on animal fitness and population dynamics.

## Supplementary Material

arac068_suppl_Supplementary_Material

## Notes

Dan Lunney, George Madani, Lachlan Wilmott, Laura Schmertmann, Caroline Marschner, Graeme Finlayson, Cristina Fernandez, Chandra Diamant, and Rob Wheeler all assisted in the field and other aspects of the project. We thank Katie Wat for assisting with the data analysis and Pushpinder Matta for the analysis of nitrogen concentrations. Rob, Judy and Doug Frend, Sam Clift and Kate Wilson, and Mark Howes from Shenhua Watermark all supplied access to their properties.
