# Supplementary material for: Patch quality and habitat fragmentation shape the foraging patterns of a specialist folivore
Source: Behav Ecol. 2022 Jul 17;33(5):1007–17. doi: 10.1093/beheco/arac068 (PMC9639584; doi:10.1093/beheco/arac068)
Supplement: arac068_suppl_Supplementary_Material [file arac068_suppl_supplementary_material.docx]

Least-Cost Paths


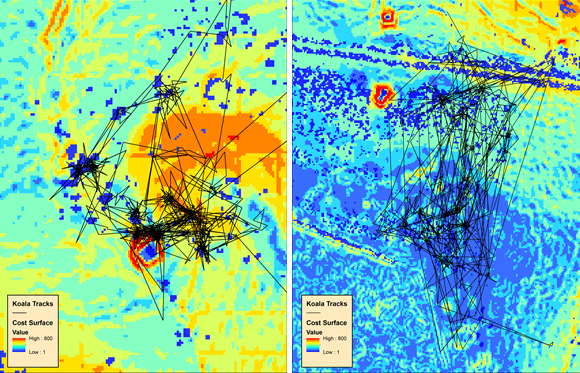


Figure S1. Example cost layer for highly fragmented habitat (left) and more contiguous habitat (right). Black lines represent two individual koalas moving in the two types of habitats
